# The Making of the Modern Practitioner

**Published:** 1911-10-28

**Authors:** 


### THE MAKING OF THE MODERN PRACTITIONER.

### THE RESULTS OF GASTRIC OPERATIONS.

Into the brain of the modern practitioner is packed away an enormous amount of information and knowledge; and that store, together with tact and address in making the best use of it, constitutes his stock-in-trade as a professional man who has a living to earn. Now, this knowledge has been acquired in various ways. To begin with, a great deal of it is obtained during student days by instruction from teachers, more or less competent to deliver the truth so far as it has been revealed to them; by the reading of text-books, some good, some bad; by attending lectures; by oral and practical clinical instruction in the wards; and even from examiners. Then in after days come larger opportunities for observation and reflection during the tenure of resident hospital appointments, combined with the chance, maybe, of working under some acute thinker or some deft surgical craftsman, and of gaining experience from the association. Then in practice, whatever kind he may enter, the doctor remains a student all his days, constantly adding to the fund of accumulated knowledge and experience which he subsequently puts at the disposal of his patients. By regular reading, also, he keeps—or at least should keep—himself abreast of the times, neither rushing in to adopt every ephemeral fad of the moment nor neglecting the real solid advances in medical and surgical science which are perpetually being made.

### A Sisyphean Task.

Unfortunately for the leisured ease of the medical profession, this store of information so laboriously acquired is subject to perpetual leakage. That is to say, much of what has been acquired in the various ways we have indicated, after a longer or shorter time ceases to be of value, and is even an actual detriment. The cargo of the mind is constantly being jettisoned as no longer useful, to be replaced by new facts, new methods, new devices, new experience. In some departments of medical work this is more noticeable than in others; in all the process is continually going on to a greater or less extent. And since the advances and novelties referred to are very largely, though not entirely, due to the activities of specialists, the task of the general practitioner in keeping track of all of them is indeed a difficult one. Let it not be supposed for a moment that the general practitioner can leave these matters to specialists. He may not need to know how to do a gastro-enterostomy or a dissection of the Gasserian ganglion; but he must know, if he is to fulfil his duty, what conditions such measures are devised for and to what degree they are successful. The instances just given are surgical, but the principle applies just as much to vaccine therapy, *x*-rays, tuberculin treatment, and every other speciality.

The practitioner who endeavours to keep himself well informed in such matters, to replenish in fact the stores of his accumulated knowledge and to reject that which has become no longer true and useful, has to wade through enormous numbers of lengthy disquisitions in the medical press. Many of these are very badly composed, difficult to understand, and also totally valueless; a few of them are published with motives very far from honourable. The result is that articles of real value become overlooked, because they are overlaid by masses of rubbish. Nevertheless, in the long run any new device which proves definitely valuable may be trusted to come eventually to the front, though the process may be unduly prolonged for the reason just given. Gradually such discoveries or improvements in medical art and science are more and more removed from the regions of controversy, and become incorporated in the current teaching of the text-books and the medical schools. The next generation of students takes them for granted as fundamental principles of knowledge, and sooner or later the public also accepts them as part of the due order of nature.

### Appendicitis.

Appendicitis furnishes a case in point. Here is a disease, now a household word, whose name was practically unknown until twenty years ago; whose symptoms were first described by a little band of surgeons, with Sir Frederick Treves as their leader so far as this country is concerned; whose correct treatment was long in dispute, and, indeed, is even yet not settled beyond all possibility of reconsideration or dissent. The student and the house surgeon of to-day become familiar, almost to the point of boredom, with cases of appendicitis. The public has taken interest in the disease and is fully alive to its dangers and its operative treatment. Society, so it was said a few years ago, was even inclined to cultivate the disease as a fashionable mode when the late King was operated on for it on the very eve of what should have been his Coronation. Practitioners, too, are for their part pretty fully informed on the subject; they have all met cases in their own practice, and they have all heard or read innumerable papers dealing with the (supposed) causes, recognition, and proper treatment of this complaint.

Gastro-enterostomy is another operation which seems to stand some risk of becoming popular with the over-introspective section of the well-to-do classes; and it is a subject upon which the modern practitioner must be prepared to stand the most searching cross-examination from dyspeptic patients. This operation up to three or four years ago was practically confined to consulting surgeons: and a good many of them, even among those attached to large hospitals, had little experience of it and of its limitations and its indications. Nowadays its technique can be laid down on definite and dogmatic lines, and many country practitioners who are keen on surgery undertake it with great success. Still the fact remains that there are still only a few men who have qualified themselves to speak from personal experience of a large number of cases, operated upon a sufficient length of time for an adequate survey of the end-results to be possible. And for the present the profession at large must rely upon the statistics of these fortunate surgeons for guidance as to the true scope of the operation, its dangers, and its triumphs.

Among those who have performed this operation an exceedingly large number of times is Mr. L. A. Bidwell, and his writings on the subject have always been distinguished by a broad-mindedness and a sanity which render his utterances especially authoritative. In a recent paper* he deals exhaustively with the results of gastro-enterostomy for gastric and duodenal ulcer. The purely medical treatment of these common and formidable ulcerations has held the field for generations; but lately the surgeons have claimed that operative treatment is safer and surer, apart altogether from the question of perforation, in which latter case it is freely admitted on all hands that immediate operation affords the only hope. Mr. Bidwell's experience extends to the large number of 181 cases of ulcer thus treated; and his conclusions are instructive, because they are founded upon a rational and logical examination of his extensive series of cases.

### End-results of Gastro-enterostomy.

Simple gastro-enterostomy, he says, is practically certain to be followed by permanent success in all the cases where there is cicatricial contraction of the pylorus or hour-glass contraction of the stomach. In the case of an ulcer situated either just below or just above the pylorus, the operation of gastro-enterostomy, combined with occlusion or division of the pylorus, is likely to be completely successful. When the ulcer is situated in the stomach at any distance from the pylorus, the operation of gastro-enterostomy by itself, even although combined with occlusion of the pylorus, is practically useless; excision of the ulcer at the same time as the gastro-enterostomy is here the proper line of treatment. The performance of gastro-enterostomy on patients who are suffering from hyperchlorhydria, producing spasm of the pylorus, and have no ulcer either in the stomach or duodenum, is not only unnecessary, but is often harmful and leaves the patient in a worse position than before, to say nothing of subjecting him to an operative risk which is most certainly not negligible.

Duodenal ulcer, according to this author, is not often permanently cured by medical treatment; and, moreover, is likely to prove fatal sooner or later, either by profuse hæmorrhage, by perforation, or by producing cicatricial contraction. It should be a rule, Mr. Bidwell considers, to operate on all cases of duodenal ulcer in which the condition can be diagnosed, and it must be an invariable practice to combine the operation with occlusion of the pylorus. In cases of gastric and duodenal ulcer where only one severe hæmorrhage has occurred it is best to wait some weeks before operating; and when gastro-enterostomy is performed the ulcer itself must be treated by excision, ligature, or pressure. When, however, repeated hæmorrhages occur an operation has to be done at once to save life; though it has to be borne in mind that an operation thus performed on a very anæmic patient is not devoid of risk. The prognosis, however, without operation is even more unfavourable than with it. Gastro-enterostomy should always be done in these cases; and when the ulcer is well away from the pylorus it should be excised. If the ulcer is on the posterior surface and adherent to the pancreas, a purse string suture should be passed all round its edge and tied tightly.

### The Economic Aspects.

Without following in detail the statistics with which these conclusions are buttressed, it is clear that so large an experience, collated with such care, must command respect and attention. Nor is it for medical men alone that the paper has an interest. It would be difficult to put before a hospital governor or subscriber any professional article of this highly technical nature which shows more clearly how and why the voluntary hospitals are spending such large sums of money. One hundred and eighty-one cases of this comparatively recent and complicated abdominal operation by one hospital surgeon! It is no wonder that operating theatres and their upkeep bulk so largely in the budgets of all hospitals that are kept abreast of the times. We feel confident that those hospital boards which occasionally wish to curtail expenditure proposed by medical and surgical staff committees will easily realise that modern equipment is essential if proper progress is to be made when they consider results such as those considered in detail above. At the same time it has to be admitted that surgeons and other specialists are often unreasonable in their demands for expensive equipment the value and utility of which is doubtful; and that one or two active and extravagantly-minded members of a hospital staff often succeed in getting their more sensible colleagues to vote for unnecessary expenditure. Such cases must, of course, be decided on their merits; all we are concerned at present to point out is that the golden mean between false economy and gross extravagance, though difficult to hit off, should be secured by friendly co-operation between the honorary medical staff and the committee of every well-administered voluntary hospital.

Exactly how it is best done depends on many circumstances, and no absolute rule can be even suggested. A very excellent way, if the right man can be secured, is for lay boards and committees to submit the proposals of a medical staff for expensive novelties to the judgment of some senior practitioner of shrewdness, ability, and common-sense; and to be guided by his advice. But in practice it is extremely difficult to find combined in one individual both long experience of men and medicine, full sympathy with the progress and aims of up-to-date specialism, tact in reconciling conflicting interests, and ability to discriminate between those new devices which are likely to be valuable and those which are likely to be merely extravagant and ephemeral.

* *West London Medical Journal*, October 1911, p. 265.

